# Supplementary material for: Constructing stage-structured matrix population models from life tables: comparison of methods
Source: PeerJ. 2017 Oct 26;5:e3971. doi: 10.7717/peerj.3971 (PMC5660883; doi:10.7717/peerj.3971)
Supplement: Supplemental Information 1 [file peerj-05-3971-s001.docx]

# Supplemental Material

| File | Description |
| --- | --- |
| table1.xlsx | Spreadsheet consisting of the parameters shown in Table 1 |
| plotfigure1.m | MATLAB script. Plots figure 1 using parameters in “table1.xlsx” |
| plotfigure2.m | MATLAB script. Plots figure 2 using parameters in “table1.xlsx” |
| plotfigure3.m | MATLAB script. Plots figure 3 using parameters in “table1.xlsx” |
| plotfigure4.m | MATLAB function. Plots figure 4 using parameters in “table1.xlsx” |
| analysis1.m | MATLAB script. Creates results used for Figures 5-16. Using parameters in “table1.xlsx”  Uses “earlymature.m” and “delaymature.m” |
| analysis2.m | MATLAB script. Creates Table 5  Uses “IterativeMethod.m” |
| earlylmature.m | MATLAB function. Calculates population growth rate and generation time using parameters in “table1.xlsx”. This function is used in “analysis1.m” |
| delaymature.m | MATLAB function. Calculates population growth rate and generation time using parameters in “table1.xlsx”. This function is used in “analysis1.m” |
| IterativeMethod | MATLAB function. Calculates population growth rate using the iterative method and parameters in “table1.xlsx”. This function is used in “analysis2.m” |
